# Sensitized Yb^3+^ Luminescence in CsPbCl_3_ Film for Highly Efficient Near‐Infrared Light‐Emitting Diodes

**DOI:** 10.1002/advs.201903142

**Published:** 2020-01-21

**Authors:** Ayumi Ishii, Tsutomu Miyasaka

**Affiliations:** ^1^ Graduate School of Engineering Toin University of Yokohama 1614 Kurogane‐cho, Aoba Yokohama Kanagawa 225–8503 Japan; ^2^ JST PRESTO 4‐1‐8 Honcho Kawaguchi Saitama 332‐0012 Japan

**Keywords:** energy transfer, lead halide perovskites, light‐emitting diodes, near‐infrared, Ytterbium

## Abstract

Near‐infrared (NIR) light emitting diodes (LEDs) with the emission wavelength over 900 nm are useful in a wide range of optical applications. Narrow bandgap NIR emitters have been widely investigated using organic compounds and colloidal quantum dots. However, intrinsically low charge mobility and luminescence efficiency of these materials limit improvement of the external quantum efficiency (EQE) of NIR LEDs, which is far from practical applications. Herein, a highly efficient NIR LED is demonstrated, which is based on an energy transfer from wide bandgap all inorganic perovskite (CsPbCl_3_) to ytterbium ions (Yb^3+^) as an NIR emitter doped in the perovskite crystalline film. High mobility of electrically excited carriers in the perovskite crystalline film provides a long carrier diffusion and enhances radiative recombination of an emission center due to minimized charge trapping losses, resulting in high EQE value in LEDs. The NIR emission of Yb^3+^ at around 1000 nm is found to be sensitized by CsPbCl_3_ thin film with a photoluminescence quantum yield over 60%. The LED based on Yb^3+^‐doped CsPbCl_3_ film exhibits a high EQE of 5.9% with a peak wavelength of 984 nm, achieved by high carrier transporting ability and effective sensitized emission property in the solid‐film structure.

## Graphical abstract

Highly efficient near‐infrared (NIR) light emitting diodes (LEDs) with external quantum efficiency (EQE) of 5.9% generated by effective sensitization of Yb^3+^ luminescence in CsPbCl_3_‐based perovskite thin film are demonstrated. The highest EQE ever reported for thin film NIR LEDs with emission beyond 900 nm provides a new possibility for future optical applications.

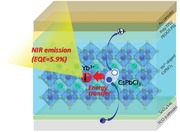

Near‐infrared (NIR) light emitting diodes (LEDs) enable a wide range of applications including night‐vision devices, optical communication, biomedical imaging, and medical treatments.^1^, ^2^, ^3^ The NIR emitters based on organic compounds (including metal complexes) and colloidal quantum dots (QDs) have been widely investigated.^4^, ^5^, ^6^, ^7^, ^8^, ^9^, ^10^ However, LED using these materials lack in sufficient level of external quantum efficiency (EQE) substantially due to low carrier mobility and trap‐assisted carrier recombination in the materials. And also, intrinsically low luminescence efficiency leaves these NIR emitting materials far from practical applications. In organic compounds, photoluminescence quantum yield (PLQY) decreases with increase in emission wavelength due to the energy gap law, mainly a consequence of increased thermal‐vibration coupling with rapid excited state quenching.^11^ Therefore, organic NIR LEDs, especially with emission wavelengths over 900 nm, exhibit poor performance with EQE less than 0.5%.^12^, ^13^, ^14^ In the case of inorganic colloidal QDs, which are emerging as promising LED materials in view of their tunable luminescence, high quantum efficiency in NIR emission can be obtained with materials prepared by solution process. For instance, lead chalcogenide QDs such as PbS and PbSe exhibit PLQYs greater than 50% in a medium of solution.^15^, ^16^, ^17^, ^18^ Nevertheless, solid‐state films of colloidal QDs for optical device applications undergo strong luminescence self‐quenching due to interdot coupling leading to carrier trapping and exciton dissociation that competes with radiative recombination.^19^ Although some attempts to prevent self‐quenching in films, such as incorporation into a polymer matrix and capping with organic ligands or inorganic shells, have been reported,^20^, ^21^, ^22^ such thick and low conductive polymer films require high external voltage for LED operation that increases power consumption.

Lead halide perovskites exhibit significant potential for applications in LEDs because of their high color purity and a narrow full‐width at half‐maximum (FWHM) over the entire visible light spectrum, as well as their low‐cost solution processing without high‐temperature treatments.^23^ After halide perovskite LEDs was first reported in 2014 by Tan et al.,^24^ EQEs of visible light emission have increased from below 1% to over 20%,^25^, ^26^, ^27^, ^28^ which is comparable to those of the best organic LEDs in the visible wavelength region. Although tuning the optical bandgap of lead halide perovskites is achieved by substitution of A‐site cation (e.g., Cs^+^, CH_3_NH_3_^+^, and HC(NH_2_)_2_^+^), and halogen ion (Cl^−^, Br^−^, and I^−^), pure lead‐based perovskite LEDs only emit below 800 nm. To improve the emission wavelength over 900 nm, some researchers adapted Sn^2+^ instead of Pb^2+^ in perovskite LEDs.^29^ Qiu et al. demonstrated mixed Pb–Sn halide perovskites LEDs which could yield efficient NIR emissions from 850 to 950 nm with the best EQE of 5.0% at 917 nm^30^ while Sn‐based perovskite materials suffer from fast degradation in ambient conditions due to the oxidation of Sn^2+^ to Sn^4+^. Highest EQE of NIR‐LED has been obtained by Vasilopoulou et al. who synthesized colloidal QDs of Ag_2_S@SiO_2_ embedded in an organo lead halide perovskite matrix. With batch‐to‐batch variability of monodispersed QDs in device performance, EQE could reach 16.98% with peak emission at 1397 nm,^31^ which is 100 times higher than that of a device without using the perovskite matrix.

In this report, we show a method to enhance EQE of NIR emitting device, not based on QDs but using a solution‐processed film of all‐inorganic perovskite (CsPbCl_3_) both as a sensitizer to NIR‐emitting ytterbium ions (Yb^3+^) and a high‐mobility matrix, to the level close to 6%. As compared with QDs or organic semiconductor‐based LEDs, a bulk crystalline film of perovskite provides a long carrier diffusion length and enhances radiative recombination of an emission center due to minimized charge trapping losses,^32^ resulting in higher EQE value in LEDs.

Yb^3+^ shows a NIR emission band at around 1000 nm assigned to the ^2^F_5/2_→^2^F_7/2_ transition of the inner‐shell 4f orbitals, which has been recognized as attractive materials for optoelectronic application in NIR region such as LEDs, lasing, and displays.^33^ However, light absorption ability of Yb^3+^ itself is significantly smaller (ε = 1–100 dm^3^ mol^−1^ cm^−1^) than that of organic dyes (ε = 10^4^–10^5^ dm^3^ mol^−1^ cm^−1^) because the electronic transitions of lanthanide ions are electric dipole forbidden (Laporte forbidden) transitions.^34^ To enhance NIR emission of Yb^3+^, sensitization, namely energy transfer from suitable donors with high absorption coefficient (as in this study) in colloidal nanocrystals (e.g., Cd or Pb chalcogenide) and in the form of Yb^3+^ complexes with organic ligands, have been attempted so far.^35^, ^36^, ^37^, ^38^, ^39^, ^40^ Unfortunately, their NIR PLQYs were still much small (<≈10%) compared to visible light emitting analogues, which prevents practical applications for NIR optoelectric devices.

It was recently found that photoluminescence of Yb^3+^ in nanocrystals of CsPbCl_3_ can exhibit a quantum yield over 100% due to quantum cutting type sensitization mechanism by using CsPbCl_3_ as a wide band gap sensitizer.^41^ This phenomenon suggests possibility to design an NIR‐emitting device based on sensitization of Yb^3+^ with CsPbCl_3_. Here, we report successful fabrication of a highly efficient NIR‐emitting device using a solution‐processed crystalline film of CsPbCl_3_ as a sensitizer and Yb^3+^ as an emitter doped into CsPbCl_3_, forming a composition of Yb^3+^‐doped CsPbCl_3_ (Yb^3+^:CsPbCl_3_). Ensuring high charge carrier mobility and balanced charge injection in the film form of solid‐state sensitizer, LED based on the Yb^3+^:CsPbCl_3_ film enabled intense NIR emission at 984 nm with significantly enhanced EQE of 5.9%, which is the new type NIR LEDs without using QDs.

The Yb^3+^‐doped CsPbCl_3_ thin film was fabricated by a multistep solution‐process (see the Experimental Section for details of film preparations). 1 m PbCl_2_ in dimethyl sulfoxide (DMSO) containing YbCl_3_ at 0.01–0.1 m was spin‐coated onto a SnO_2_ coated quartz substrate. After being dried at 90 °C for 15 min, 0.07 m CsCl methanol solution was spin‐coated onto Yb^3+^ doped PbCl_2_ film and continuingly heated at 250 °C for 5 min. This process was repeated for five times to obtain a 120‐nm‐thick perovskite thin film. As displayed in **Figure** **1**a inset image, the film is highly transparent (93% transmittance as shown in Figure S1 in the Supporting Information) to show the logo printed on the substrate. Stoichiometric range of Yb^3+^ is 1.0–9.1 mol% in CsPbCl_3_, which is determined by the solution stoichiometry. Further doping Yb^3+^ prevents a film formation of CsPbCl_3_. In Figure 1a, X‐ray diffraction (XRD) patterns of undoped CsPbCl_3_ and Yb^3+^ doped CsPbCl_3_ (Yb^3+^(9.1 mol%):CsPbCl_3_) films show diffraction peaks at 15.8^o^, 22.5^o^, 26.6^o^, 32.1^o^, 35.9^o^, 39.4^o^, and 46.0^o^, assigned to (100), (110), (111), (200), (210), (211), and (220) crystal planes of CsPbCl_3_, respectively, which confirms the formation of cubic‐phase perovskite crystal without detectable crystalline impurities. Here, we found that 0D Cs_4_PbCl_6_ phase increased when PbCl_2_ film slowly reacted with CsCl solution (Figure S2, Supporting Information),^42^ which strongly affects the luminescent property of the film as described later. Negligible shifts of the XRD reflections are observed with Yb^3+^ doping in this concentration range. On the other hand, X‐ray photoelectron spectroscopy (XPS) measurements revealed chemical interactions between CsPbCl_3_ and Yb^3+^. As shown in Figure 1b, CsPbCl_3_ exhibits Cl 2p XPS band at 197.9 eV, which is broadly observed in a higher energy side by doping Yb^3+^. The higher energy band, which essentially implies reduced electron density on chloride ions, corresponds to Cl^−^ bonded to Yb^3+^. It means that a part of Pb^2+^ was replaced to Yb^3+^.^41^, ^43^ It is supported by the result that Yb 4d XPS bands were slightly shifted to a lower energy side in CsPbCl_3_ compared with YbCl_3_.

**Figure 1:**
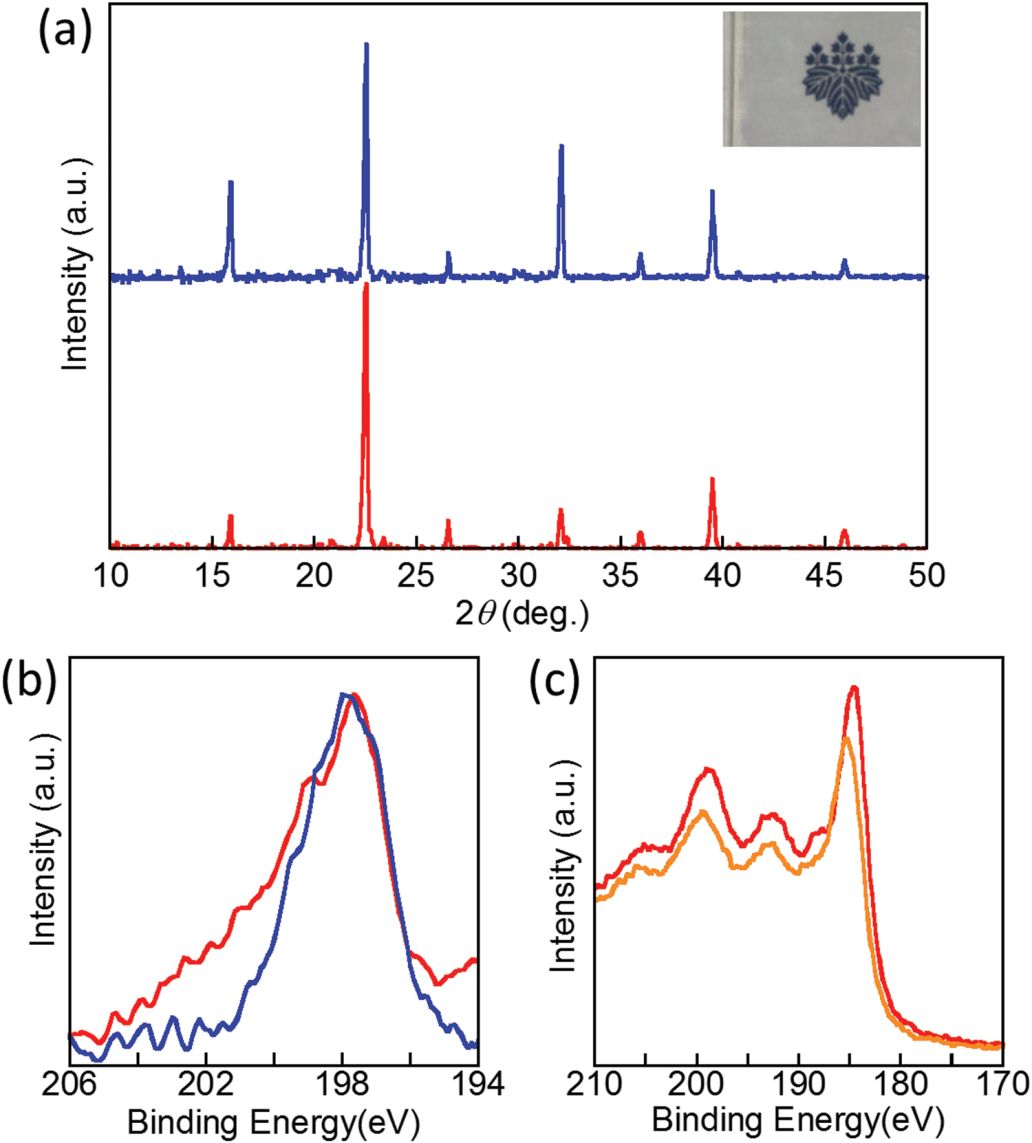
a) XRD patterns of CsPbCl_3_ (blue) and Yb^3+^:CsPbCl_3_ (red) films (λ = 1.54 Å). Inset shows transparency of an Yb^3+^:CsPbCl_3_ film coated on a glass substrate with the university logo. b) Cl 2p and c) Yb 4d XPS bands of CsPbCl_3_ (blue), Yb^3+^:CsPbCl_3_ (red) films, and YbCl_3_ (orange).

**Figure** **2**a shows photoluminescence (PL) and the excitation spectra of CsPbCl_3_ and Yb^3+^:CsPbCl_3_ film. The undoped CsPbCl_3_ film shows a band‐edge excitonic emission at 415 nm with the emission lifetime of 23.8 ns (Figure S3a, Supporting Information).^44^, ^45^ The photoluminescence quantum yield of CsPbCl_3_ film is less than 0.1% due to lower exciton binding energy in perovskite crystalline films than that in low dimensional nanocrystals. It is noteworthy that this emission from CsPbCl_3_ (415 nm) is completely quenched by doping of Yb^3+^ at 9.1 mol% to CsPbCl_3_. On excitation of CsPbCl_3_ at 320 nm, CsPbCl_3_:Yb^3+^ film exhibits strong emission at 984 nm, which is assigned to the ^2^F_5/2_→^2^F_7/2_ transitions of Yb^3+^. Such strong NIR luminescence is hardly observed by direct excitation of Yb^3+^ at 980 nm even using a strong diode laser (≈430 W cm^−2^). The excitation spectrum monitored at 984 nm matches the absorption spectrum of CsPbCl_3_. These results indicate that the NIR luminescence of Yb^3+^ is effectively enhanced by the energy transfer from CsPbCl_3_ to Yb^3+^ even in the polycrystalline film structure. The lifetime of this emission from Yb^3+^ in CsPbCl_3_ is estimated as a single emission component of 1.68 ms (Figure S3b, Supporting Information), indicating that Yb^3+^ is uniformly dispersed in CsPbCl_3_ film.

**Figure 2:**
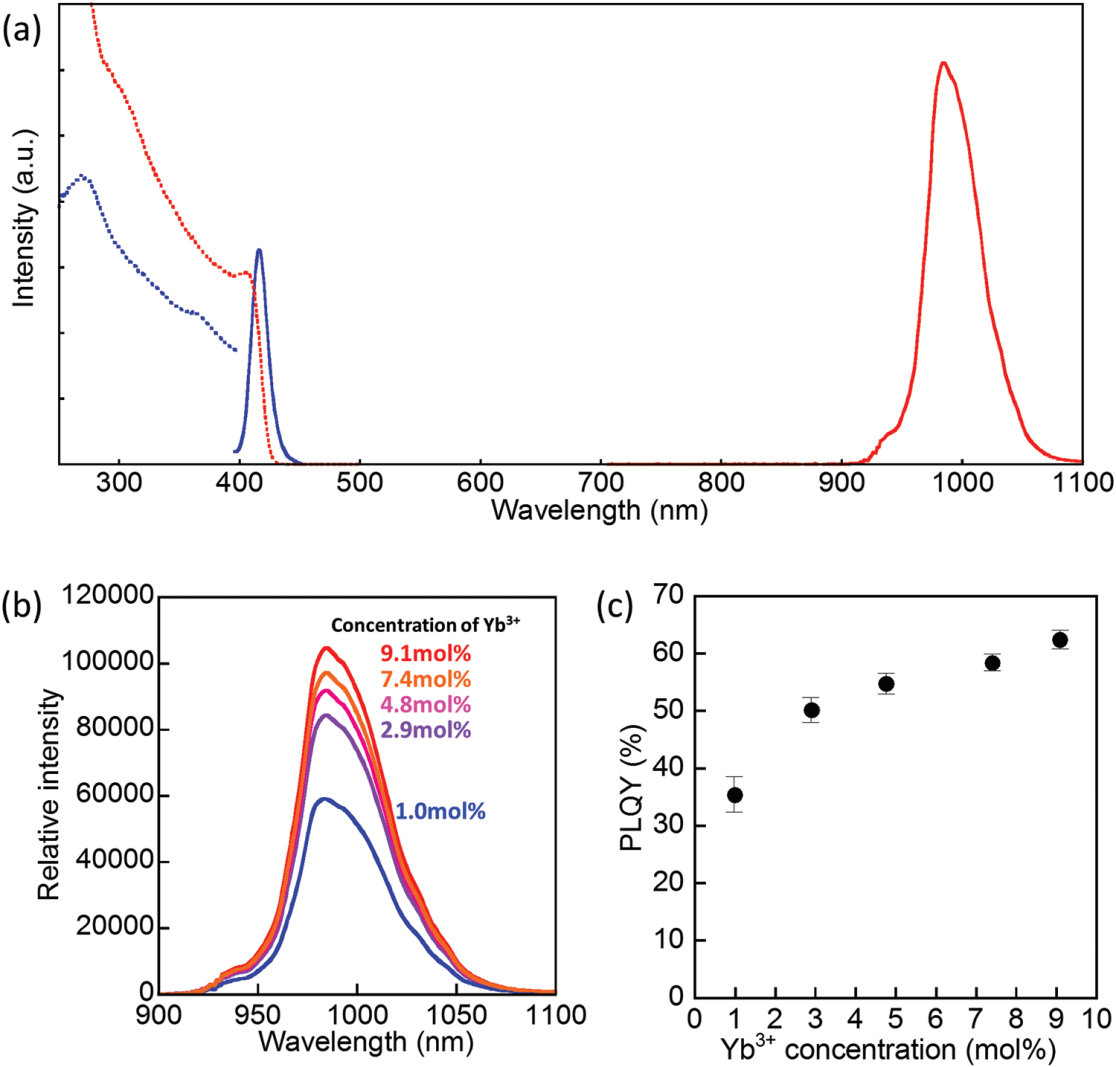
a) Photoluminescence (solid line) and the excitation (dotted line) spectra of CsPbCl_3_ film (blue) and Yb^3+^(9.1 mol%):CsPbCl_3_ film (red) (λ_ex_ = 300 nm, λ_det_ = 415 nm (CsPbCl_3_), and 984nm (Yb^3+^:CsPbCl_3_)). b) Concentration dependence of Yb^3+^ on photoluminescence spectra (Yb^3+^ concentrations are defined as [Yb^3+^]/([Yb^3+^]+[Pb^2+^])). c) NIR PLQYs plotted as a function of Yb^3+^ molar concentration.

The stoichiometry of cesium lead halides, CsPbCl_3_ or Cs_4_PbCl_6_, is important for the energy transfer process to Yb^3+^. It is known that a crystal structure of Cs_4_PbX_6_ phase is very different from that of CsPbX_3_, resulting in the large bandgaps and strong excitonic absorption for Cs_4_PbCl_6_.^46^ Cs_4_PbCl_6_ film obtained in this study shows a broad emission band around 350 nm with the lifetime of 1.14 ns (Figures S3a and S4, Supporting Information).^47^, ^48^ This emission from Cs_4_PbCl_6_ is still observed in Yb^3+^:Cs_4_PbCl_6_ while the emission intensity of Yb^3+^ is negligibly small (Figure S4, Supporting Information), which means that the energy transfer from Cs_4_PbCl_6_ to Yb^3+^ hardly occurs. In Yb^3+^:CsPbCl_3_, the NIR emission intensity increased with increasing Yb^3+^ concentration from 1 mol% to 9.1 mol% with respect to Pb^3+^ (Figure 2b). The absolute PLQY measured using an integrating sphere increased from 31.0% up to 62.3% (Figure 2c). Although a condensed film structure generally causes nonradiative relaxation processes of photoexcited materials due to the surface and lattice defects as compared with nanocrystals, we could observe the highest NIR PLQY over 60% with the thin film structure as achieved by the efficient energy transfer with a quantum‐cutting process.

We fabricated a perovskite NIR LED using the highest emissive Yb^3+^(9.1 mol%):CsPbCl_3_, employed in a device structure; transparent conducting oxide (TCO) glass/SnO_2_ (10 nm)/8‐quinolinolato lithium (Liq) (≈2 nm)/Yb^3+^:CsPbCl_3_ or CsPbCl_3_ (120 nm)/poly*N, N′*‐bis(4‐butylphenyl)‐*N, N′*‐bis(phenyl)‐benzidine (poly‐TPD)/poly(3,4‐etylenedioxythiophene):poly(styrenesulfonate) (PEDOT:PSS) (60 nm)/Au (80 nm) (see the Experimental Section for details of device fabrications). **Figure** **3**a displays cross‐sectional view of the device structure observed by scanning electron microscopy (SEM). The energy diagram of the perovskite LED is shown in Figure 3b. The valence bands of CsPbCl_3_ and Yb^3+^:CsPbCl_3_ were estimated by ultraviolet photoelectron spectroscopy (UPS) in air atmosphere (Figure S5, Supporting Information), in which the energy position of CsPbCl_3_ is slightly lowered by doping of Yb^3+^. The conduction band levels were determined by Tauc plot analysis with a bandgap of 3.0 eV (Figure S6, Supporting Information). SnO_2_ nanoparticles were used as the electrotransport/hole‐blocking layer (ETL/HBL). Liq was deposited on the SnO_2_ coated cathode to accelerate the electron injection from SnO_2_ to the perovskite layer. CsPbCl_3_ and Yb^3+^:CsPbCl_3_ emissive layers are then formed on the SnO_2_/Liq layer. PEDOT:PSS and poly‐TPD work as a hole‐transport/electron‐blocking layer. Finally, Au (80 nm) was evaporated on the top as the anode. To estimate charge injection balance, we measured the current density versus voltage (*J–V*) characteristics of electron‐injection‐only device (TCO/SnO_2_/Liq/Yb^3+^:CsPbCl_3_/Au and TCO/SnO_2_/ Yb^3+^:CsPbCl_3_/Au) and hole‐injection‐only device (TCO/Yb^3+^:CsPbCl_3_/poly‐TPD/PEDOT:PSS/Au) (Figure S7, Supporting Information). According to the *J–V* results, more balanced charge injection occurs due to an improvement of electron injection by inserting a thin Liq layer between SnO_2_ and CsPbCl_3_, which was supported by observation of very weak electroluminescence in the Liq‐free device. **Figure** **4**a shows the *J–V* curves of the complete devices. The turn‐on voltage of the Yb^3+^:CsPbCl_3_ device is shifted to the higher side as compared with that of the CsPbCl_3_ device as a result of an increase in the hole injection barrier between poly‐TPD and perovskite layers.

**Figure 3:**
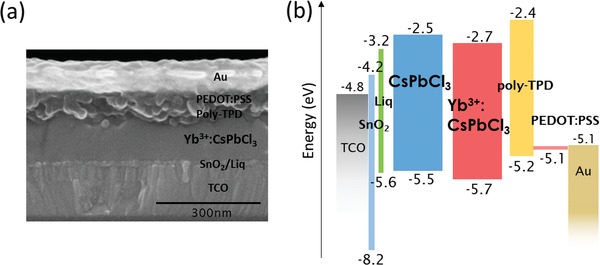
a) Cross‐sectional SEM image of the Yb^3+^:CsPbCl_3_ based LED. b) Energy diagram of the charge transfer materials in LED.

**Figure 4:**
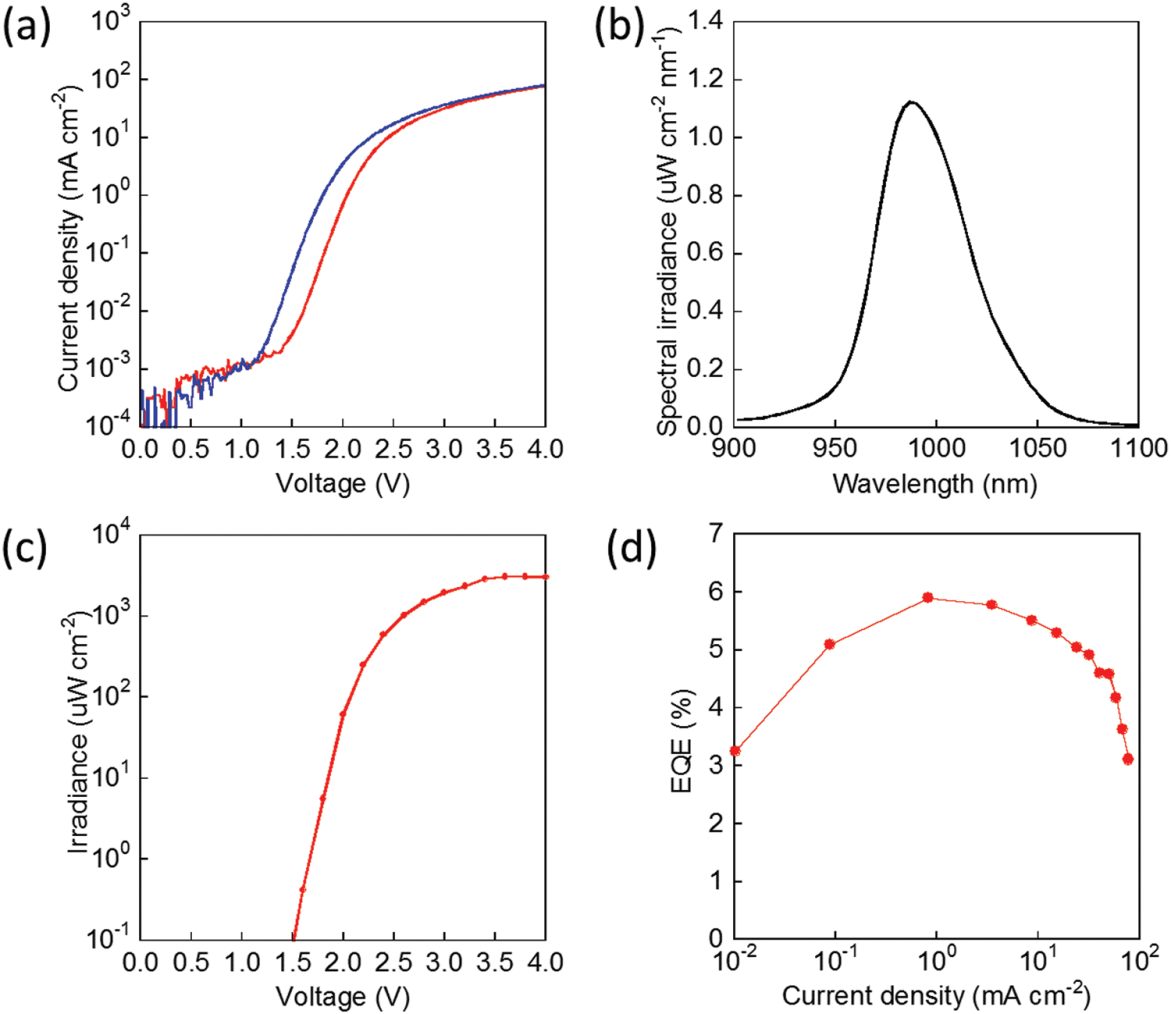
a) Current density–voltage curves for CsPbCl_3_ (blue) and Yb^3+^:CsPbCl_3_ (red) based LEDs. b) NIR electroluminescence spectrum (applied voltage, 2 V), c) irradiance–voltage, and d) EQE–current density characteristics of the Yb^3+^(9.1 mol%):CsPbCl_3_ based LED.

In the CsPbCl_3_ based device, weak electroluminescence is broadly observed around 400 nm and 670 nm (Figure S8, Supporting Information), which assigned to a direct transition from the conduction band to the valence band (CB→VB) in CsPbCl_3_ and a transition from the conduction band of CsPbCl_3_ to the HOMO level of PEDOT:PSS, namely CB(CsPbCl_3_)→ HOMO(PEDOT:PSS), respectively. The weak intensity of electroluminescence from CsPbCl_3_ (EQE is not detected in our system) is because of the excition dissosiation with high charge transport in the perovskite crystalline film. The Yb^3+^:CsPbCl_3_ based device showed a strong NIR electroluminescence at 984 nm under an applied voltage of 2 V, as shown in Figure 4b, while no luminescence was observed in the visible wavelength region. It suggests that the energy transfer from electrically excited CsPbCl_3_ to Yb^3+^ effectively occurs as well as the case of photoexcitation, which is enhanced by effective carrier diffusion to an emission center composed of Yb^3+^ partially replaced in a CsPbCl_3_ lattice.

The irradiance–voltage curve of Yb^3+^:CsPbCl_3_ device is shown in Figure 4c. The Yb^3+^:CsPbCl_3_ device has a low turn‐on voltage of ≈1.6 V, which was enabled by the high‐mobility of perovskite film and the efficient carrier injection from HTL and ETL, yielding a high irradiance of 3100 µW cm^−2^ at 3.6 V. The EQE value of the Yb^3+^:CsPbCl_3_ device reaches a maximum of 5.9% at 0.827 mA cm^−2^ (Figure 4d) which is the highest EQE ever reported for thin film type NIR LEDs capable of emission beyond 900 nm.^30^ The NIR electroluminescence half‐life time (*T*_50_) in air condition under applying a constant current (0.827 mA cm^−2^) has reached 58 h (Figure S9, Supporting Information). We confirm that highly performance for NIR LEDs is achieved by constriction of the unprecedented NIR emissive system composed of the perovskite thin film and Yb^3+^.

In conclusions, we have demonstrated the fabrication of highly NIR luminescent CsPbCl_3_ perovskite films doped with Yb^3+^ and the bright NIR LEDs based on it. Yb^3+^:CsPbCl_3_ film shows a strong NIR luminescence through the efficient energy transfer from perovskite to Yb^3+^, leading to accomplishment of the highest NIR PLQY, over 60%, in thin‐film structures. The Yb^3+^:CsPbCl_3_ based LEDs also exhibit a bright electroluminescence around 1000 nm with EQEs up to 5.9%, which was achieved by high carrier transporting ability and effective sensitized emission property in the solid‐film structure. The material and method of the Yb^3+^:perovskite‐based NIR LEDs reported here open up its applications to night‐vision devices, optical communication, biomedical imaging, and medical treatments.

## Experimental Section

##### Perovskite Film Preparation

The perovskite film was fabricated by a multistep solution‐process. 1 m PbCl_2_ (Sigma‐Aldrich) in DMSO containing YbCl_3_ (Sigma‐Aldrich) at a stoichiometric range of 0–9.1 mol% was spin coated onto glass substrates (quartz for optical measurements and TCO for LED device) at 3000 rpm for 30 s. After being dried at 90 °C for 15 min, 0.07 m CsCl methanol solution was spin coated onto PbCl_2_ film at 3000 rpm for 30 s and continuingly heated at 250 °C for 5 min. This process was repeated for five times to obtain the ideal perovskite films. The obtained perovskite films were rinsed with isopropanol and dried 250 °C again for 5 min. The Cs_4_PbCl_6_ phase was obtained when PbCl_2_ film slowly reacted with CsCl solution by spin coating at 2000 rpm for 30 s.

##### Device Fabrication

Transparent conducting oxide (TCO, an ITO–ATO composite) glass (15 Ω sq^−1^, Geomatic Co., Ltd.) was cleaned sequentially with acetone, isopropanol, and deionized water by putting them under sonication for 10 min in each of the solvents, and then dried by blowing N_2_ gas. The TCO glass was finally treated under oxygen plasma for 10 min. The SnO_2_ blocking layer was coated on TCO glass by spin‐coating a diluted aqueous solution of SnO_2_ nanoparticle (15% in H_2_O colloidal dispersion, Alfa Aesar, was diluted to be ≈2% before being used) with 30 min of drying at 150 °C. Liq was deposited on the SnO_2_ coated TCO substrate by thermal evaporation under vacuum at pressures of below 5 × 10^−5^ Pa. After that, the perovskite films were deposited under ambient atmosphere as described in the perovskite film preparation section. On top of the perovskite films, poly‐TPD dispersed in toluene (4 mg mL^−1^) was spin‐coated at 1000 rpm for 60 s and heated at 150 °C for 20 min. PEDOT:PSS in toluene solution was also coated at a spin speed of 3000 rpm and heated at 150 °C for 15 min. Then, Au layer was finally deposited on the top as counter electrode by vacuum evaporation. The active area of the device was 3 × 3 mm^2^.

##### Characterizations

XRD patterns were measured by D8 DISCOVER (BrukerAXS K. K.) with Cu Kα radiation under the operation condition of 40 kV, 40 mA to determine the crystal structure of perovskite. XPS was performed using a Kratos Axis Ultra delay‐line detector equipped with a monochromatic Al Kα X‐ray source (1486.6 eV). SEM measurements were performed with SU8000 (Hitachi High‐Technologies Co.) to check the thickness and morphology of the layers. Ionization potential ultraviolet photoelectron spectroscopy (UPS) in the air was estimated by a photoemission yield spectrometer (AC‐3, Riken Keiki Co., Ltd.). Photoluminescence spectra were recorded on FP‐8600 spectrometer (JASCO Corporation). PLQY for NIR emission was measured using an absolute photoluminescence quantum yield measurement system (Quantaurus‐QY plus, Hamamatsu Photonics K. K.). The emission decay curves were acquired using a Quantaurus‐Tau (Hamamatsu Photonics K. K.) with excitation of LED or xenon flash lamp with a band‐path filter. Photocurrent density–voltage (*J−V*) curves in forward bias conditions were measured by a computer‐controlled digital source meter (Keithley 2450). Electroluminescence characteristics were recorded with a Flame spectrometer coupled with a cosine corrector to collect signals from 180° field of view (Ocean Optics). Lambertian emission was assumed in the calculation of EQE and irradiance.^49^ Peak EQE was determined as the number of forward‐emitted photons to the number of injected electrons.

## Conflict of Interest

The authors declare no conflict of interest.

## Supporting information

Supporting Information
