# Supplementary material for: Sensitized Yb3+ Luminescence in CsPbCl3 Film for Highly Efficient Near‐Infrared Light‐Emitting Diodes
Source: Adv Sci (Weinh). 2020 Jan 21;7(4):1903142. doi: 10.1002/advs.201903142 (PMC7029626; doi:10.1002/advs.201903142)
Supplement: Supplementary file 1 — Supporting Information [file ADVS-7-1903142-s001.pdf]

Copyright WILEY-VCH Verlag GmbH & Co. KGaA, 69469 Weinheim, Germany, 2020.

ADVANCED SCIENCE
Open Access

Supporting Information

for *Adv. Sci.,* DOI: 10.1002/advs.201903142

Sensitized Yb^{3+} Luminescence in $CsPbCl_3$ Film for Highly Efficient Near-Infrared Light-Emitting Diodes

Ayumi Ishii and Tsutomu Miyasaka**

Copyright WILEY-VCH Verlag GmbH & Co. KGaA, 69469 Weinheim, Germany, 2020.

Supporting Information

Sensitized Yb^{3+} Luminescence in $CsPbCl_3$ Film for Highly Efficient Near-Infrared Light-Emitting Diodes

Ayumi Ishii, and Tsutomu Miyasaka**

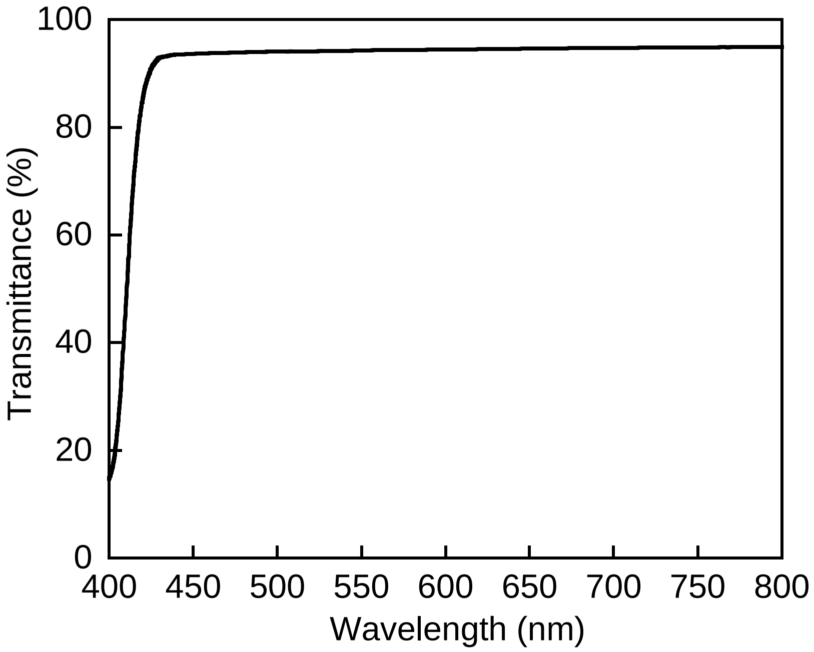

Figure S1. Transmittance spectrum of Yb^{3+}:$CsPbCl_3$ film on quauz glass substrate.

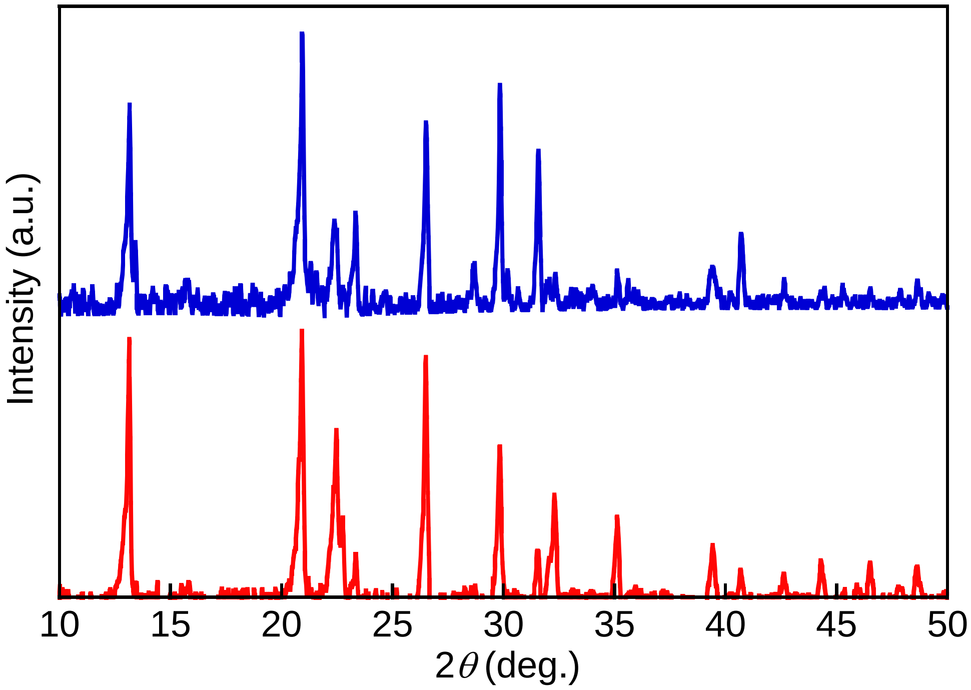

Figure S2. XRD patterns of Cs_4PbCl_6 (blue) and Yb^{3+}:Cs_4PbCl_6 (red) films (λ =1.54 Å). The peak at 22.5^{o} is assigned to $CsPbCl_3$ phase slightly contained in Cs_4PbCl_6.

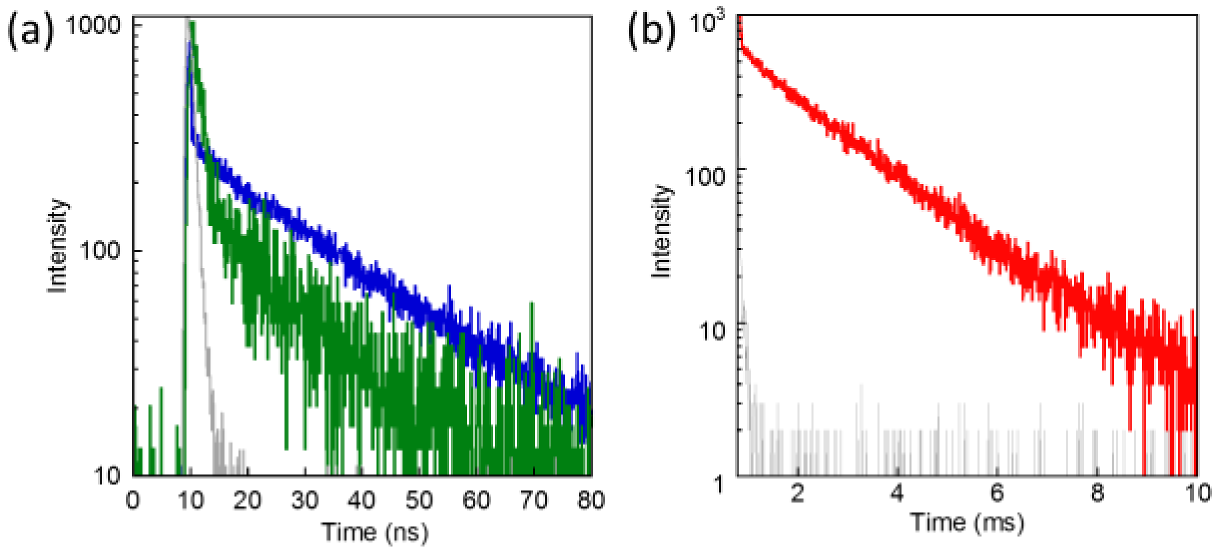

Figure S3. Luminescence decay curves of a) $CsPbCl_3$ (blue, λ_{ex} = 280 nm, λ_{det} = 415 nm), Cs_4PbCl_6 (green, λ_{ex} = 280 nm, λ_{det} = 350 nm) , and b) Yb^{3+}:$CsPbCl_3$ (red, λ_{ex} = 340 nm, λ_{det} = 984 nm). The longer lifetime component observed in Cs_4PbCl_6 is consistent with that of $CsPbCl_3$. The gray line shows the instrument response function.

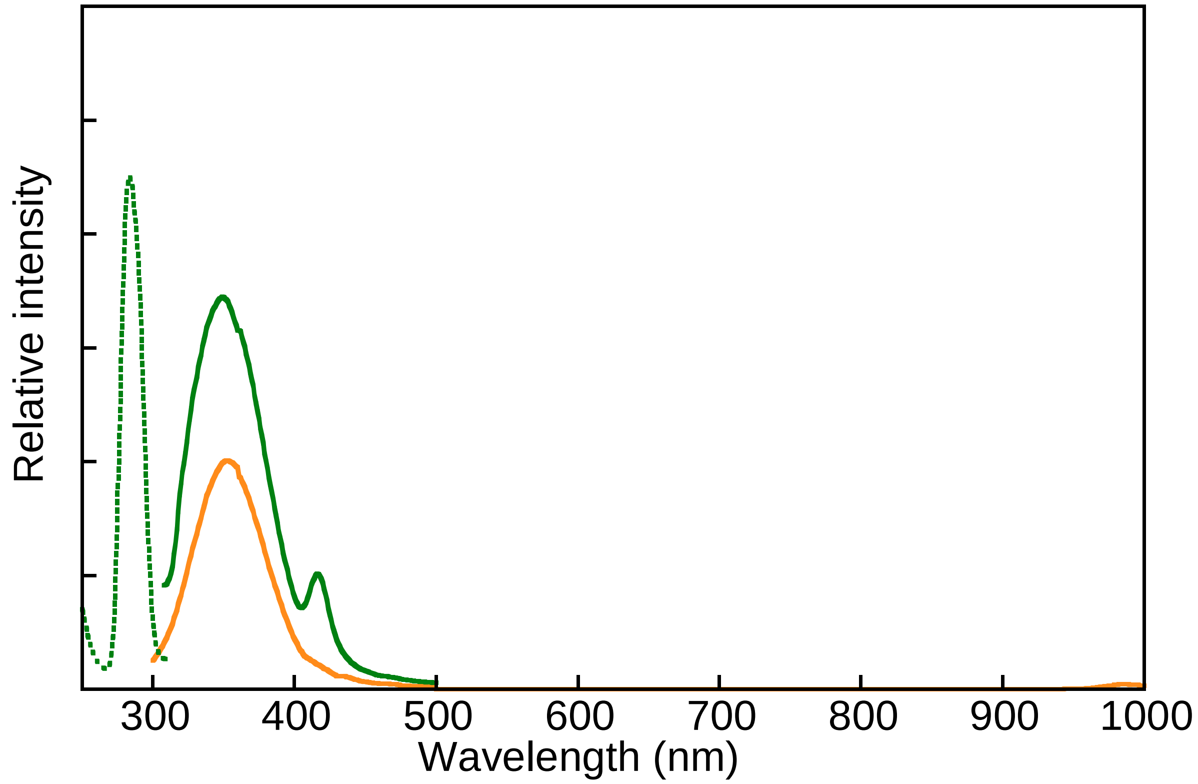

Figure S4. Photoluminescence and the excitation spectra of Cs_4PbCl_6 (green) and Yb^{3+}(9.1mol%): Cs_4PbCl_6 (orange) films (λ_{ex} = 280nm, λ_{det} = 350 nm). The emission observed at 415 nm is originated from $CsPbCl_3$.

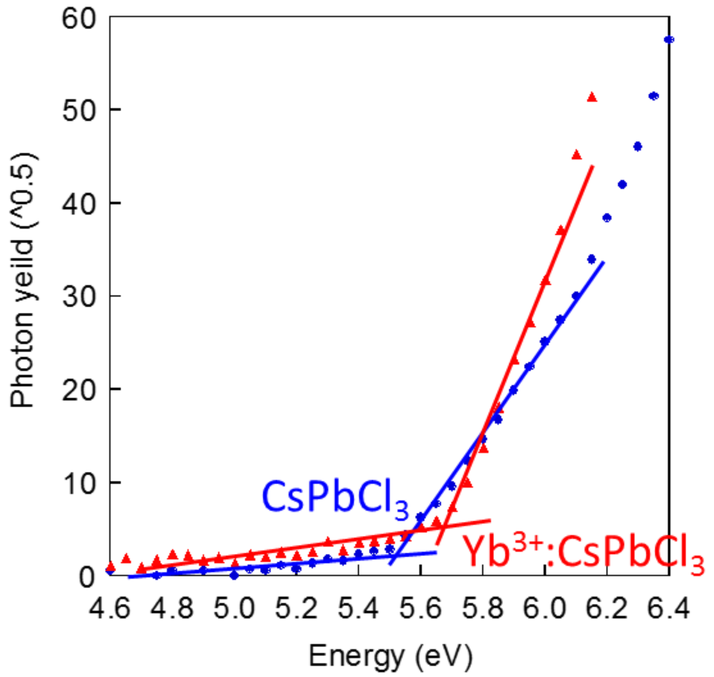

Figure S5. UPS spectra observed in air atmosphere of $CsPbCl_3$ (green) and Yb^{3+}(9.1mol%): $CsPbCl_3$ (red) films.

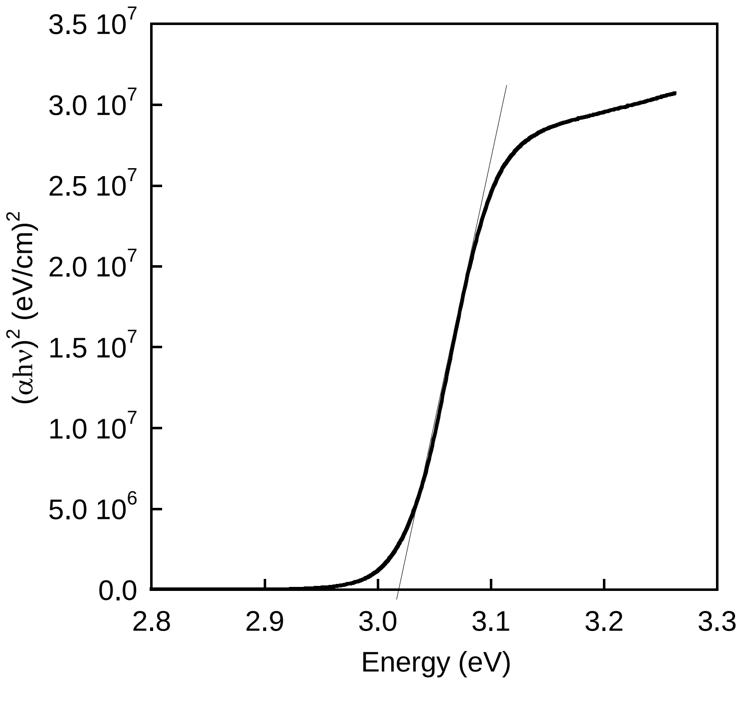

Figure S6. Tauc plot analysis for the direct allowed transition estimated by transmittance spectrum of Yb^{3+}:$CsPbCl_3$ film.

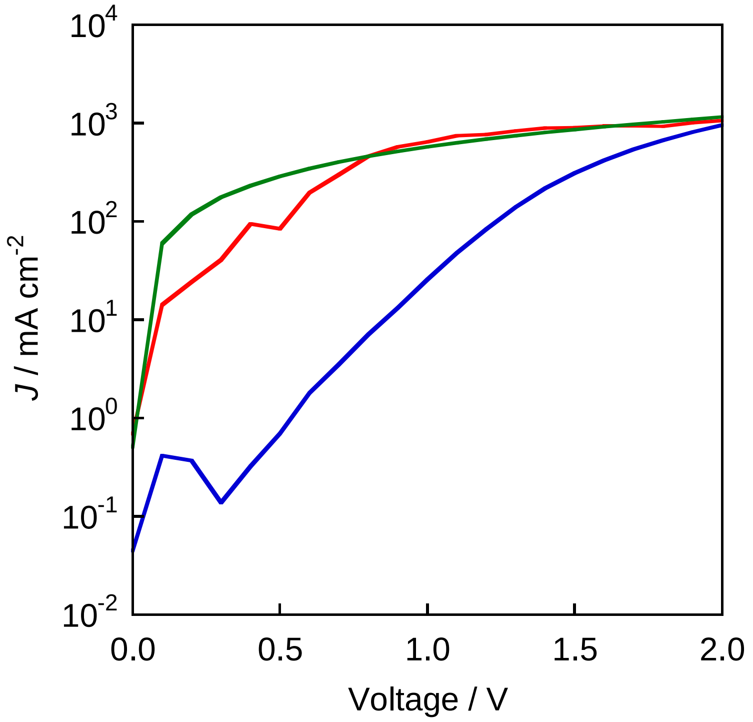

Figure S7. Current density-voltage curves of electron-injection-only devices (TCO/SnO_2/Liq/Yb^{3+}:$CsPbCl_3$/Au (green) and TCO/SnO_2/ Yb^{3+}:$CsPbCl_3$/Au (blue)) and hole-injection-only device (TCO/Yb^{3+}:$CsPbCl_3$/poly-TPD/PEDOT:PSS (red)).

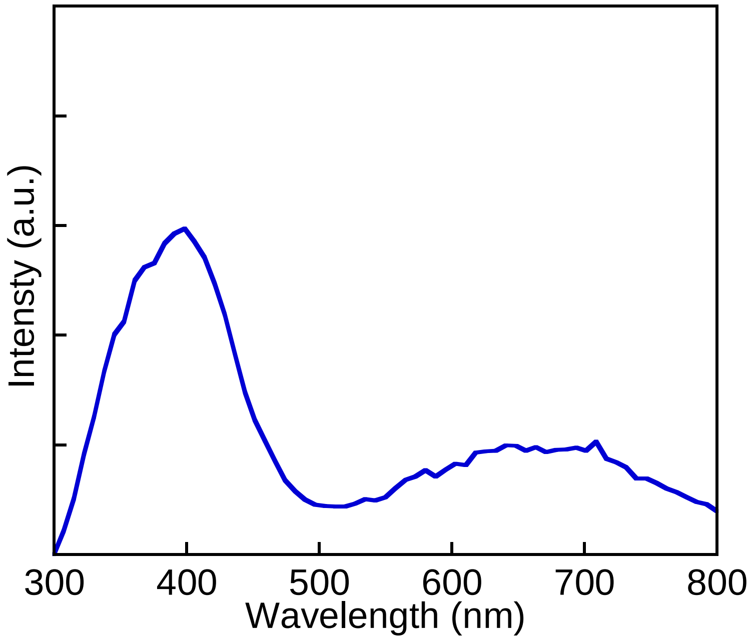

Figure S8. Electroluminescence spectrum of the $CsPbCl_3$ based LED (applied voltage, 4V).

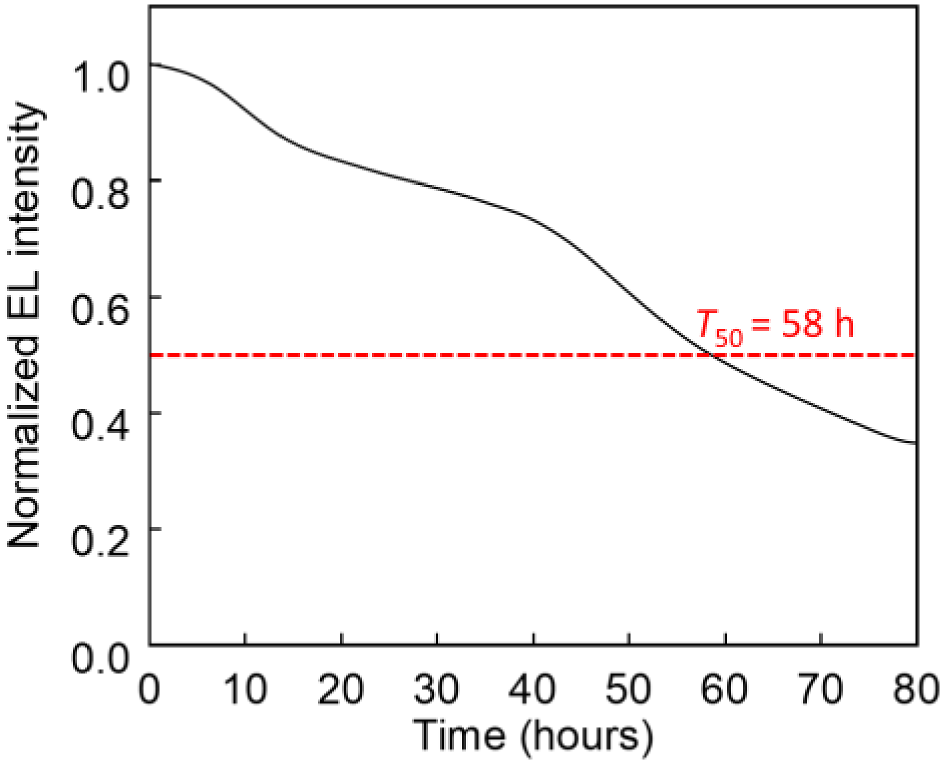

Figure S9. Operational stability lifetime of the Yb^{3+}(9.1mol%):$CsPbCl_3$ based LED, performed in air condition under applying a constant current (0.827 mA cm^{-2}).
